# Effect of Neck Muscle Vibration Prior to Motor Learning on Short-Latency SEP Peak Amplitudes and Motor Performance

**DOI:** 10.3390/brainsci15121311

**Published:** 2025-12-05

**Authors:** Alexandre Kalogerakis, Paul Yielder, Hailey Tabbert, Bernadette Murphy

**Affiliations:** Faculty of Health Sciences, Ontario Tech University, Oshawa, ON L1G 0C5, Canada; alexandre.kalogerakis@ontariotechu.net (A.K.); paul.yielder@ontariotechu.ca (P.Y.); hailey.tabbert@ontariotechu.net (H.T.)

**Keywords:** Electroencephalography (EEG), Somatosensory Evoked Potentials (SEPs), body schema, proprioception, motor acquisition, motor learning, muscle spindles, neural processing

## Abstract

*Background/Objectives*: Neck muscle vibration alters neural processing, sensorimotor integration, and proprioception in healthy adults. Significant differential changes in the N18 and N24 somatosensory evoked potential (SEP) peak amplitudes, coupled with altered motor learning, occurred when completion of a force-matching task took place following neck muscle vibration. It is currently unknown if neck muscle vibration also impacts acquisition of skills from visuomotor tracking tasks, a gap this research addresses. *Methods*: A total of 25 right-handed, healthy participants were divided into vibration (age: 21.7 ± 1.89, n = 13; 8 females) (V) and no-vibration (NV) control (age: 21.2 ± 3.03, n = 12; 6 females) groups. The vibration was device applied over the right sternocleidomastoid and left cervical extensor muscles. The participants underwent right-median-nerve stimulation at 2.47 Hz and 4.98 Hz to elicit SEPs. A total of 1000 sweeps were recorded and averaged using an ANT Neuro Waveguard 64-lead EEG cap (ANT Neuro, the Netherlands, Manufactured by Eemagine, Berlin, Germany)pre- and post-completion of a novel visuomotor tracing task (MTT). Post-acquisition, the NV group had a 10 min rest, and the V group received 10 min of vibration at 60 Hz before motor task completion, followed immediately by post-acquisition and retention 24 h after. *Results*: N18 peak: The V group exhibited a proportional amplitude increase of 19%, while the NV group exhibited a 36% decrease. There was a trend toward decreased retention in the V group. P25 showed a significant effect of time, with increases of 11% for V and 9% for NV. *Conclusions*: V resulted in N18 SEP changes post-MTT-skill-acquisition. Both groups appeared to learn, with the V trending towards less retention.

## 1. Introduction

At any given moment, the brain receives and processes multiple peripheral inputs conveying information about the body’s position in time and space. The ability to integrate these senses to enable accurate performance of a task-specific response is known as sensorimotor integration (SMI) [1,2], and it relies on accurate perception and processing of incoming sensory information [3,4,5,6]. A growing body of research is investigating the effect of altered afferent input from the neck on upper-limb SMI [7,8] joint position sense [9,10,11], and motor learning [12,13,14,15]. Longer-term changes in neck sensory input that occur with subclinical neck pain (SCNP) [12,13] or transient alterations due to changes in muscle spindle discharge induced by vibration [15] or fatigue [16,17] also lead to changes in upper-limb SMI. This work suggests that an altered afferent input impacts SMI, likely by influencing forward models needed to update the body schema and refine efference plans with precision while performing a novel motor task. While experimental fatigue represents a method of examining transient muscle spindle input leading to alterations in processing, these effects are not long-lasting and might not lead to prolonged changes [16,17]. Muscle vibration offers an outlet of transiently altering muscle spindle discharge in which prolonged effects are observed in response to ten minutes of vibration [18]. While previous studies employed different protocols of vibration ranging from whole-body [19,20,21], to neck muscle vibration [15,22,23,24], there is evidence supporting the role of neck afferents in the construction of an accurate body schema and the ability to perform subsequent tasks [6,9,25,26]. Using the sensory information from our surroundings, the nervous system constructs and constantly updates the body schema, a 3D representation of the body in space derived from shape of each segment, length, proprioceptive information, and limb configuration [5,27]. This process is reliant on accurate representation of the head and neck, and alterations may have implications for motor control and learning.

The motor-learning literature makes it evident that the cerebellum plays a key role in the process of acquiring novel skills during various stages of learning [28,29,30]. Studies using SEPs found differential changes in peaks associated with cerebellar activity in response to learning a novel task reliant on forms of proprioception such as force matching [15,31] and motor tracing [13,32]. Visuomotor tracing relies on the accurate processing of sensory input in order to refine efferent plans with precision [33]. The acquisition of visuomotor tracing skills has been shown to result in differential SEP peak activation compared to a novel force-matching task [31], suggesting that these two tasks require different processing. Previous work investigated the effects of subclinical neck pain (SCNP) [13] and fatigue [34] on the processing and acquisition of a novel tracing skill, demonstrating altered acquisition eventually leading to a decreased ability to retain the skill in the SCNP and fatigue groups compared to the controls. This demonstrates that altered sensory signals from the neck impact processing of upper-limb sensory information.

Cervical musculature contains a high density of muscle spindles [35,36], and neck inputs influence the processing of afferent information from other areas [3]. This has been supported by research demonstrating that changes in neck positioning affect upper-limb joint position sense (JPS) and arm-guided movements [9,11]. This mechanism is thought to be due to the alterations in the way that neck and head position influence the body schema [9,11,18]. However, it has been proposed that these errors may be intensified by local biomechanical mechanisms, where torque-producing muscles of the neck connecting to the upper limb and the upper fibres of the trapezius (UFTs) introduce potential confounding variables [11]. Vibration can induce illusions of tonic muscle contractions and joint rotation if visual feedback is occluded [37]. High-frequency, low-amplitude vibration over muscle bellies selectively excites the fastest-conducting 1A afferents feeding from muscle spindles [38], potentially leading to spindle discharge, causing the CNS to perceive length changes and movement [3] so long as the frequency is in the 30–100 Hz range [26,39].

When neck muscle vibration is applied to healthy individuals, it can alter JPS [3,37,40], balance [22,23,41], and cortical–cerebellar processing in response to the acquisition of a novel motor skill [15]. These findings are thought to be due to the altered muscle spindle input relayed to the central nervous system (CNS) causing transient alterations in the way that the CNS perceives proprioceptive input [26]. A previous study examined the effects of neck muscle vibration on motor skill learning in regard to a novel force-matching task heavily reliant on proprioception and force modulation [15]. It would be beneficial to investigate the effects of vibration on the learning of a novel visuomotor tracing task reliant on accurate visual cues and proprioception to conduct the task [31,33,42], as the student and working populations routinely use such skills to complete day to day workplace-specific tasks. The acquisition of a visuomotor skill has been shown to affect cerebellar processing measured via various imaging techniques [13,28,31,32,43]. There are also differences in neural processing between a force-matching task and a visuomotor task [31]. There is an understanding of how the transient alterations of vibration affect healthy individuals’ processing in response to a motor-learning task reliant on force modulation and proprioception, but there is less understanding of how vibration affects the ability to learn a task that is heavily reliant on visual cues to guide motor plans.

It was hypothesized that vibration would result in differential changes in SEP peaks reflecting cerebellar processing. The study aimed to investigate the effects of neck muscle vibration on the neural processing and motor learning of a novel visuomotor tracing task.

## 2. Materials and Methods

### 2.1. Participants

This study had an experimental pre/post design and involved 25 right-handed participants (aged: 21.4 ± 2.39, sex: 14F) recruited from the Ontario Tech University population. A total of 13 participants were randomly assigned to the vibration group (V) (aged: 21.7 ± 1.89, sex: 8F), with 12 participants assigned to the no-vibration group (NV) (Aged: 21.2 ± 3.027, sex: 6F). Pre-screening included the Edinburgh handedness inventory (EHI), administered to assess handedness and cortical preference since left-handed individuals exhibit neural-processing and laterality differences [44], and the Neck disability Index (NDI) to ensure the participants did not have neck pain, as such pain has been shown to alter SEP peak suitability involved in motor learning [45]. The neurophysiology screening checklist was also administered to ensure that the participants had no previous or current neurological diseases or history of medication and/or substance use that might alter cognitive ability or EEG analysis. The inclusion criteria required that participants score > 40 on the EHI (right-hand-dominant) and <5 on the NDI (no pain). Verbal and written consent were required prior to participation, and the study was approved by the university Ethics board (File #: 16520).

### 2.2. Somatosensory Evoked Potentials (SEPs) Stimulation Parameters

Controlled electrical stimulus with a duration of 0.1 ms was administered via a Digitimer DS7A constant current stimulator (Welwyn Garden city, UK) over the median nerve at 2.47 Hz and 4.98 Hz through bi-polar Ag/AgCl ECG conductive adhesive skin electrodes (MEDITRACE™130, Ludlow Technical Products Canada Ltd., Mansfield, MA, USA). The electrodes were placed 2–3 cm proximal to the distal crease of the wrist, with the anode placed proximally [46,47]. Stimulation at both frequencies was administered since the N30 SEP peak can be clearly identified without signal attenuation, whereas the N24 SEP peak is best visualized with the attenuation effect of a higher stimulation rate of 4.98 Hz [48]. The stimulation intensity was above the sensory and motor threshold of the median nerve, leading to a reproducible twitch of the right abductor pollicis brevis (APB) muscle, with ∼1 cm deviation from anatomical position in order to ensure the fastest-firing, type 1A fibers were stimulated [47]. We ensured that there was no pain or discomfort, as it could have affected our recorded signal [45]. A total of 1000 stimuli were administered at both frequencies as per International Federation of Clinical Neurophysiologist (IFCN) guidelines.

### 2.3. Recording Parameters

Surface electrodes were placed at non-cephalic sites in accordance with the IFCN guidelines [47,49]. Specific sites: The Erb’s point/brachial plexus electrode was placed posterior to the clavicle and referenced to an ipsilateral ear clip. An electrode was placed over the spinous process of the 5th cervical vertebra referenced to the anterior tracheal cartilage. Lastly, a contralateral ground electrode was placed over the bony prominence of the clavicle. All peripheral recording sites were thoroughly prepped with a razor, abrasive tape, and alcohol swab prior to the placement of the electrodes.

Central SEP peaks were recorded with a 64-lead ANT Neuro WaveGuard (ANT Neuro, the Netherlands, Manufactured by Eemagine, Berlin, Germany) Electroencephalography (EEG) cap, applied in accordance with the internationally standardized 10–20 system recommended by the IFCN [47,49]. This measurement system uses anatomical landmarks with specific pre-specified proportions to standardize each participant’s head. Measurements (in cm) of the circumference (to determine EEG cap size) and distance from nasion to inion of the participant’s head are taken. Following this, 50% of the nasion-to-inion number is taken to determine the placement of the Fz electrode at the vertex of the skull. To ensure accurate positioning of the cap, frontopolar and occipital electrode placement is measured to ensure there is a 10% distance above the nasion and the inion, respectively. All EEG cap electrodes were referenced to the common average reference, which is the average electrical activity across all channels of the EEG. Electrical impedances were kept below 5 kΩ and 10 kΩ for the surface EMG electrodes and 64-channel EEG cap, respectively. SEPs were recorded at two frequencies (2.47 Hz and 4.98 Hz). The 4.98 Hz frequency was used to attenuate the N30 SEP peak to enhance our ability to measure the N24 SEP peak [48]. This method was adopted based on previous research demonstrating that at lower frequencies, the N30 masks the N24, but stimulating at a higher frequency, around 4.98 Hz, has an attenuation effect on the N30, making it easier to distinguish the N24 peak, enabling more accurate measurements of amplitude changes, which are important for assessing cerebellar and S1 processing.

The peripheral and spinal SEP peak signals (N9, N11, and N13) were amplified by a gain factor of 10,000 while preprocessing using Signal^®^ software (Version 4.08, Cambridge Electronic Design, Cambridge, UK). An average of the 1000 median nerve stimuli (SEP) was taken and filtered with a 0.2–1000 Hz bandpass filter since this has been shown to produce accurate and reliant SEP waveforms [13]. The signal recorded with the EEG was amplified by a gain of 40,000 during post-processing using Advanced Source Analysis (ASA) v. 4.10.1 Software (ASA™; Hengelo, The Netherlands). The removal of artefacts, including eye blinks and EMG activity from the ocular, neck, and jaw muscles, occurred during post-processing to ensure accurate extraction of central SEP peaks without the influence of signal artefacts. The cut-off parameters for noise extraction ranged from +100 to −100 µV. Parameters for signal cleaning and filtering were employed in ASA™; these tasks consisted of removal of eye blink artefacts by selecting three or more eyeblinks. Eyeblinks are represented as positive deflections due to dipole interactions between the cornea and the electrode. The software then creates a template for removal of all eyeblink artefacts. Next, a bandpass filter with a low cut-off frequency of 0.2 Hz and high of 1000 Hz is applied. Artefact detection parameters are then selected: min −100 and max +100 µV. The final step consists of averaging the recording and setting a time base between −0.01 and 0.2.

### 2.4. Neck Muscle Vibration Protocol

**Figure 1:**
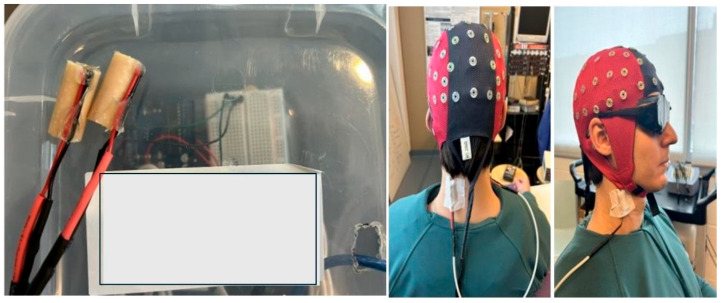
(**Left**) panel depicts the vibrators which were placed on the right SCM and left CEM; (**right**) panel shows vibrators and EEG cap with blackout goggles on a mock participant.

Vibration at 60 Hz for 10 min at an amplitude/displacement of less than or equal to 1 mm [11,15] was applied over the right sternocleidomastoid (SCM), with the electrode placed 2 cm anterolaterally and 6 cm inferior to the mastoid process, and left cervical extensor muscles (CEMs), with the electrode placed 2–3 cm lateral to the 5th-cervical-vertebra spinous process [15]. This was carried out using two small vibration motors produced by Precision Microdrives™ (Model # 310-112; London, UK) that measure approximately 10 mm in diameter and were housed in a 3D-printed casing and secured in place using Hypafix medical tape. It is imperative to ensure the devices are on the muscle in the correct anatomical location but not placing a significant amount of pressure on the area, as this may affect the amplitude and thus the receptor stimulated. The participant wore vision-occluding goggles to stop any visual feedback correcting the proprioceptive illusion (Figure 1). A custom-coded 6 pin Arduino Uno board (Arduino, Strambino, Italy) was used to operate the vibrating devices, allowing for changes in frequency and duration. A Fast Fourier Transform was employed during piloting to ensure the amplitude set on the board matched the desired frequency of 60 Hz. Pilot work has shown that 60 Hz vibration at 10 min was not sufficient to impact SEPs alone when a mental recitation task was used in place of a learning paradigm [15]. The mental recitation task was used in order to standardize the activity in between SEP recordings for isolation if vibration alone influenced the recording. As per the study, there was no change in pre-to-post SEP recording.

### 2.5. Motor-Tracing Task

**Figure 2:**
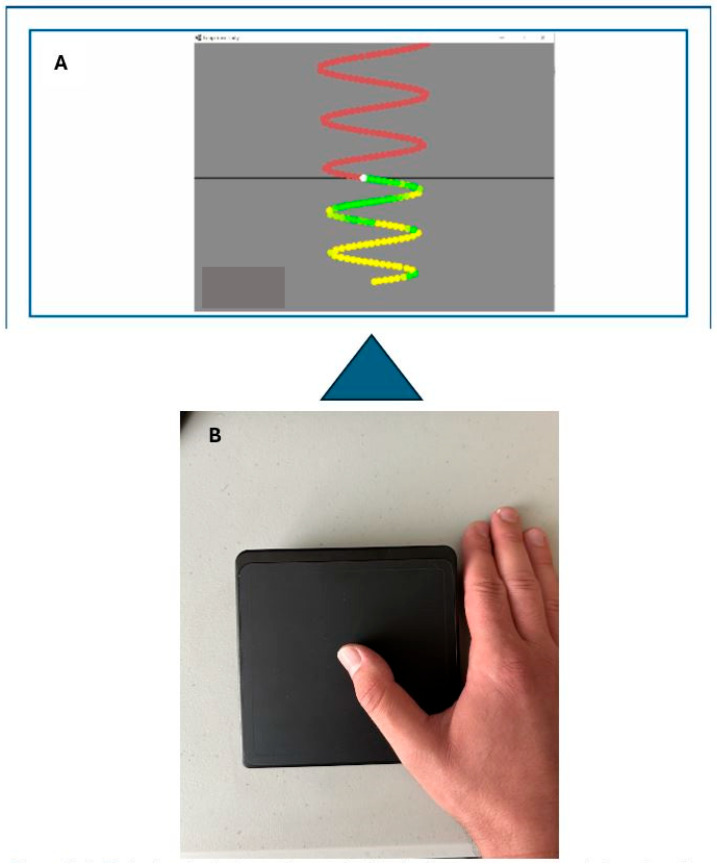
(**A**): Motor tracing task-colour coded to display green as accurate touch and yellow as inaccurate. (**B**): Participants hand set-up on the cursor pad.

In the Motor-Tracing Task (MTT), participants sat in front of a monitor and were instructed to sit comfortably in the chair. The program was run through a custom Leap Motion Unity software tool v.12 (Leap Motion, Inc., San Francisco, CA, USA) [13], which required participants to trace sequences of sinusoidal waves by moving their right thumbs on a wireless touchpad (Logitech, Inc., Fremont, CA, USA). The trace was a sine wave consisting of 500 dots per trial and varied randomly in frequency and amplitude throughout each trial. The pre and post phases consisted of 4 trials, which lasted approximately 3–4 min, while the acquisition phase was 12 trials lasting approximately 10–15 min. The participant was instructed to isolate their thumb muscle during the trace to avoid any wrist deviations and shoulder movements while performing the task (Figure 2).

### 2.6. Experimental Protocol

**Figure 3:**
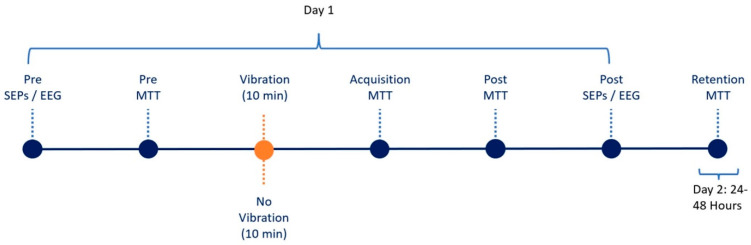
Experimental Flow Demonstrating two-day sequence where each dot reflects stage.

SEPs were recorded before and after the acquisition of the MTT skill. Following baseline recordings, the participant would begin the task, which would start with a familiarization trial, followed by a pre-acquisition block consisting of 4 trials. Participants would either be subjected to vibration or rest, with vibrators affixed for 10 min prior to the start of the acquisition phase of the task. Following acquisition, the post-acquisition phase of the task, consisting of 4 trials, was completed. Post-SEPs were recorded after the post-acquisition block. Participants returned 24–48 h after for a retention phase consisting of the last 4 trials (Figure 3).

## 3. Data Processing

### 3.1. SEP Analysis

Inclusion criteria: A stable N9 (<±20% change from pre- to post-application) was required in order to include data for subsequent analysis. A stable N9 is essential in determining if the changes observed are a result of altered neural processing and not a change in the incoming afferent volley itself due to factors such as postural changes and voluntary movement [47,50]. No participants were excluded from data analysis

SEP peaks were recorded at a sampling frequency of 2048 Hz using Cambridge Electronic Design (CED) Signal software (Version 4.08, Cambridge Electronic Design, Cambridge, UK). Peripheral SEP peaks N9, N11, and N13 were analyzed using the Signal software, and the central SEP peaks N18, N20, N24, P25, N30, and N60 were analyzed using Advanced Source Analysis (ASA). Peak amplitudes were quantified by measuring the amplitude difference between the preceding trough and the succeeding peak, and they were normalized by dividing the absolute post value by the absolute pre value. This step is implemented to prevent any individual differences from cofounding the signal, as pre-SEP values of an individual vary in amplitude. This is imperative as individual differences in aspects such as sex, height, and tissue distribution may affect nerve conduction velocity, thus acting as potential individual cofounding variables [46,47,50]. The N9 SEP peak is recorded over Erb’s point, where the brachial plexus resides, representing the ascending afferent volley post-stimulation of the median nerve.

### 3.2. Motor-Learning Analysis

Percent error was calculated using the Leap Motion Unity software tool v.12 (Leap Motion, Inc., San Francisco, CA, USA) by taking the average distance the participant deviated from the waveform, which is called the perfect trace. If the error was 100%, the participant deviated one full dot length away from the perfect trace. Mean percent error from each trial was then averaged [32]. Analysis of the motor-tracing task involved the evaluation of changes in error during the task. Each trial’s raw error percentage was averaged per given phase. For example, the pre-phase consisted of four trials; the average error percentage was taken to form the “pre average”. The same protocol was repeated for the post-phase and retention. Prior to averaging, the data was graphed in a line chart over time for visual inspection to ensure no outliers influenced the calculation. A group average was then taken, where the percent error was determined proportionately to the pre phase by dividing the pre phase by itself to ensure the impact of large individual differences would be reduced when examining the results. Post was then divided by the pre value, and retention was divided by post, in order to measure the proportional change in error.

### 3.3. Statistical Analyses

All statistical analyses were conducted with IBM SPSS version 29 (Armonk, New York, NY, USA). SEPs and MTT data were analyzed using a repeated-measures analysis of variance (ANOVA). Data met the assumptions of normal distribution as assessed using Mauchly’s test of sphericity; if violated, the Greenhouse–Geisser correction would be applied. Normalized SEP data was analyzed using a 2-way repeated-measures ANOVA with group (V vs. NV) as the between subjects’ factor and time (expressed as pre/post) as the repeated measure. MTT data consisting of normalized error rate was analyzed with a 3 × 2 repeated-measures ANOVA with group (V/NV) as the between-subjects factor and time (pre, post, and retention) as the within-subjects repeated measure. Pre-planned contrasts were used to compare baseline to post-acquisition and post-acquisition to retention. Statistical significance was set at *p* ≤ 0.05 for all tests. For the motor performance data, Mauchly’s test of sphericity was used to test sphericity, and corrections were made using the Greenhouse–Geisser if the result was not spherical. Lastly, partial eta squared (ηp^2^) values are reported with 0.01, 0.06, and 0.14 equal to small, medium, and large effect sizes for ANOVAs, respectively [51,52].

## 4. Results

All 25 participants were included in the analysis as none violated the N9 stability criterion of ±20% put in place to ensure all changes observed were due to neural processing and not postural and movement changes. However, two participants, one in each group, had a large number of deleted sweeps during the 4.98 Hz recording. Therefore, the N24 data sets for these two participants were excluded from the data and statistical analyses.

### 4.1. Time–Group Interactions

#### N18 SEP Peak

There was a significant time–group interaction for the N18 SEP peak, where amplitude increased 19.3% in the V group and decreased by 35.6% in NV (F_(1,23)_ = 5.046, *p* = 0.035, ηp^2^ = 0.180).

There was no significant time–group interaction between N11 (F_(1,23)_ = 0.658, *p* = 0.425, ηp^2^ = 0.028), N13 (F_(1,23)_ = 1.720, *p* = 0.203, ηp^2^ = 0.070), N20 (F_(1,23)_ = 0.649, *p* = 0.429, ηp^2^ = 0.027), N24 (F_(1,23)_ = 0.583, *p* = 0.453, ηp^2^ = 0.026), N30 (F_(1,23)_ = 1.357, *p* = 0.256, ηp^2^ = 0.056), and N60 (F_(1,23)_ = 0.944, *p* = 0.341, ηp^2^ = 0.039).

### 4.2. Time Effects

#### P25 SEP Peak

There was a significant effect of time for the P25 SEP peaks, where amplitude increased by 11.7% in the V group and ↑ 7.5% in the NV group (F_(1,23)_ = 6.992, *p* = 0.014, ηp^2^ = 0.233). There were no significant time effects for the other SEP peaks.

**Figure 4:**
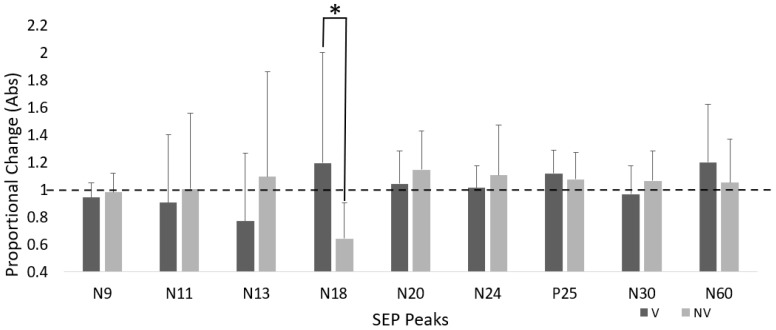
Proportional change in SEP peak amplitude for both groups. Dotted line represents baseline. Error bars are SD (* *p* < 0.05).

See Table 1 for SEPs data (Figure 4).

### 4.3. Motor Performance Accuracy

**Figure 5:**
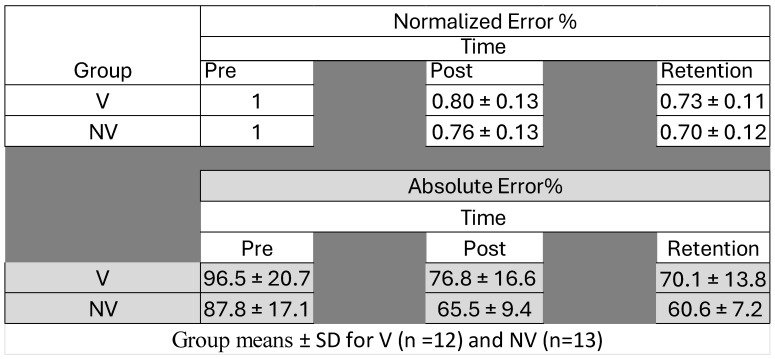
Error rates (normalized and absolute) for both groups pre- and post-acquisition and upon retention (24–48 h afterwards).

**Figure 6:**
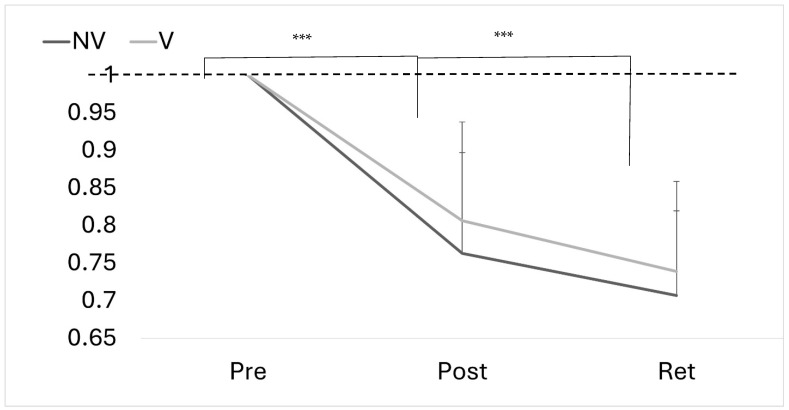
Proportional change in absolute error at pre, post and retention for both groups. Error bars represent SD. Dotted line represents baseline (*** *p* < 0.001).

There was an overall effect of time for motor performance (F_(2,23)_ = 58.710, *p* < 0.001, ηp^2^ = 0.719), with no significant time–group interaction (F_(2,23)_ = 0.0005, *p* = 1.000, ηp^2^ = 0.019) (Figure 5 and Figure 6).

## 5. Discussion

This study was the first investigation of the effect of neck vibration on visuomotor task learning and short-latency SEP amplitudes. Neck muscle vibration resulted in differential changes in SEP peak amplitudes of the N18 SEP peak, with no significant differences in motor skill performance between the two groups. While the motor performance differences did not reach significance, it may be worth investigating the long-term effects of vibration. This could be indicative of the effect of vibration on short-term processing in specific neural networks associated with cerebellar input.

### 5.1. N18 SEP Peak

Our findings indicate that vibration increased the amplitude of the N18 SEP peak compared to the decrease in the controls. This peak is thought to correspond to multiple subcortical structures in the midbrain pontine region reflecting and modulating inhibitory activity of the inferior olivary tract entering the cerebellum via the inferior peduncle [53,54,55,56]. Recent research suggested that the neural generators of N18 appeared to be localized near the ventral intermediate nucleus in the zone incerta of the pre-lemniscal radiation [53], a white matter region within the post-subthalamic region [57]. However, the researchers did not record cerebellar activity, so its involvement is unclear. Prior research has suggested that the N18 peak originates in lower medullary nuclei [58] and then branches off from the DCML and the cuneocerebellar tract into the inferior olivary–cerebellar tracts [56]. Although the current literature lacks consensus, the generally accepted generators are the multiple aforementioned subcortical structures. Researchers using randomized multiresolution scanning (RAMUS) of SEP data have suggested that the N18 peak is widely distributed over the brainstem, with the majority of activity stemming from the lower medulla and dorsal column [59]. Recent research demonstrated a significant increase in N18 amplitude post-motor acquisition in those subjected to neck muscle vibration during acquisition, while the NV group exhibited a decreased N18 peak. Increases in the N18 peak have been seen in previous research applying vibration to healthy individuals learning a force-matching task more heavily reliant on proprioceptive input [15]. The N18 increase in the V group may indicate a heavier reliance on proprioception to learn the task to offset the distorted body schema imposed by vibration. The decrease in N18 in the NV group coincides with previous studies [13,31,32]. The decrease has been suggested to be reflective of reliance on visuomotor cues to complete a task resulting in decreased inhibition in the networks comprising the N18 [31]. The findings may suggest differential SMI changes at the level of the cerebellum leading to a potential re-weighting of stimuli to complete a given task since the vibration group may have more proprioceptive influences guiding their motor plans to complete the task, whereas the control groups can rely solely on visuomotor cues. Past research has shown that acquisition of novel force-matching task skills increases the N18 peak amplitude, which is hypothesized to be an increase in proprioceptive information moving from the upper limbs into the cerebellum [31]. The increase in N18 seen in our V group may also reflect an upregulation of proprioceptive signaling post-vibration, since vibration stimulates 1a afferents and creates changes in perception of head and neck positions, which remain distorted for several hours. This may force participants to rely more on feedback processing of proprioceptive cues to complete the task, as the illusory effects of vibration would distort feedforward processing. Overall, our findings coincide with others’ results suggesting that vibration may affect proprioception mediated by the illusions of muscle movement.

### 5.2. N24 SEP Peak

The N24 peak is thought to be reflective of the earliest cortical somatosensory processing and may be localized along the linkage between the cerebellum and the primary somatosensory cortex (S1) [47,50,60,61]. Our study findings contrast with previous studies investigating motor learning and N24. We found slight non-significant increases, which contrast with the Andrew’s work, where the N24 SEP peak decreased after motor tracing in healthy individuals [13,32]. However, these studies used two cephalic electrodes instead of a high-density EEG cap, which enabled the placement of a single electrode at a recommended location. It is possible that the use of the cap increased the variability of the N24 measures. The findings reported by Ambalavanar and colleagues align with the observed non-significant increase in the N24 peak of a similar amplitude while using a 64-EEG electrode cap [31]. Another possibility may be that since the time of Andrew’s work, the use of smartphones has increased among young adults [62]. It may be that due to the near-constant use of the thumb to scroll and interact with screens, the tracing task has become less novel to participants, resulting in smaller changes in specific SEP peaks. Research has shown that a decrease in cerebellar output may reflect decreasing inhibition, which can be associated with later stages of motor learning [29,30]. This has been observed in prior SEP research, where a decrease in N24 amplitude occurred following motor acquisition of a force-matching task skill in healthy controls [15], and this decrease correlated with the ability to learn and retain a novel motor skill. The absence of significant time effects may also indicate that visuomotor ability did not impact this pathway in the same way or to the same degree as the force-matching task, which was more reliant on accurate proprioception.

### 5.3. P25 SEP Peak

Although no time–group interaction was observed, there was a significant effect of time for the P25 SEP peak, with an increase for both groups in response to the task, irrespective of vibration exposure. P25 is thought to play a role in the processing of hand tactile input directed to the somatosensory cortex [50,63,64]. This increase aligns with prior research in which the thumb was used to either modulate forces [15,31] or trace a waveform. The pattern observed here may represent increased processing of the sensory inputs sent to the somatosensory cortex in response to the acquisition of a thumb-dominant motor-tracing task skill.

### 5.4. Motor Performance

The motor-tracing task relied heavily on visuomotor information, which requires the participant to constantly revise efferent plans in order to adapt to the changing environment of the task [33]. Both groups were able to learn the task, but there were no significant group differences. This has been seen in previous studies assessing neural processing associated with motor skill learning [15,31]. Interestingly, the idea of recalibrating the body schema has been shown in the literature to rely on adaptation of multiple sensory subsystems, which may be a long-term manifestation in certain cases [65,66,67]. Permanent modifications lead to profound differences in neural networks in order to adapt [65]. While prior studies on body schema mainly investigated the effects of elective plastic surgery such as breast reduction or augmentation [66], functional movement disorders [67], limb-lengthening surgery [65], and brain damage with left unilateral spatial neglect [68], it is clear that the CNS adapts to various conditions or procedures by forming differential connections between neural networks mediated by the body schema. While not as extreme as the aforementioned examples, the short-term changes in sensory input from the neck induced by vibration demonstrated differential changes in processing of the N18 SEP peak in response to a novel motor-training task. While vibration exposure has been shown to alter sensorimotor function in a dose–response relationship with aftereffects lasting several hours, it could be that these transient alterations are not robust enough to affect the long-term manifestations of the body schema. Previous work demonstrated that SEP changes precede changes in motor cortex output measured using TMS [69]. Furthermore, the same study showed that over the longer term, SEP changes were better correlated with motor learning. While the decrease in error observed in our study was not significant, this information may provide insight for future research investigating motor learning while under the influence of vibration over longer time periods to see if exposure duration (both in terms of time and/or number of sessions) affects motor performance. Previous work has shown similar trends in response to force matching in regard to a proprioception-based task where the control group trended towards learning better but not significantly [15]. While motor-tracing tasks have been shown to alter different SEP peaks relative to force-matching tasks [31], suggesting the involvement of different neural networks, they may provide insight into the more profound effect of vibration on proprioception-based tasks. Vibration has been shown to greatly impact upper-limb JPS in healthy individuals [22,23,40], which may indicate the proprioceptive illusion brought on by vibration selectively alters proprioception-based tasks such as upper-limb and cervical JPS.

## 6. Limitations

This study investigated neural processing in response to learning a novel motor task; however, the sample was recruited from the university population (18–35 years of age), so these findings cannot be generalized to those younger or older than the specified thresholds. Furthermore, the transition to hybrid or remote forms of learning may increase reliance on visuomotor tasks due to the prolonged period of use of technologies such as tablets and computers. This may have implications affecting the performance of university-aged participants, as they are more adept at navigating these tasks. The pre-screening also did not control for those who were adept at video games since this ability may translate to quicker learning of novel visuomotor tasks, potentially affecting performance metrics.

## 7. Conclusions

This study demonstrated differential changes in SEP peak amplitudes via exposure to vibration. There was an increase in activity over the region of interest thought to be associated with cerebellar input, which may reflect a greater reliance on proprioceptive input to complete the task, which may suggest that processing of identical information occurs in a different way. While both groups learned the task, a long-term investigation should be conducted to correlate the differences in the N18 between groups with the impact on motor learning. Future work should assess different frequencies and durations of vibration exposure, since the literature has not yet come up with a standardized protocol. Furthermore, assessments of a proxy measure for proprioception such as JPS should be implemented to further support proprioceptive changes. A more complex visuomotor task that represents activities of daily living or workplace demands should also be incorporated, as this may give researchers a better idea of how vibration exposure affects these areas.

## Figures and Tables

**Table 1:** Proportional changes in SEP peak amplitudes with SD of vibration vs. control group.

Proportional Changes in SEP Peak Amplitude	Group		Time–Group *p*-Values	Effect of Time *p*-Values
SEP Peaks	V	NV		
**N9**	0.94 ± 0.11	0.98 ± 0.13	0.361	0.033
**N11**	0.90 ± 0.49	1.00 ± 0.55	0.425	0.522
**N13**	0.77 ± 0.49	1.09 ± 0.76	0.203	0.564
**N18**	1.19 ± 0.80	0.64 ± 0.25	0.035	0.513
**N20**	1.04 ± 0.23	1.14 ± 0.28	0.429	0.120
**N24**	1.01 ± 0.16	1.10 ± 0.36	0.453	0.338
**P25**	1.11 ± 0.17	1.07 ± 0.19	0.572	0.014
**N30**	0.96 ± 0.20	1.06 ± 0.21	0.256	0.708
**N60**	1.19 ± 0.42	1.05 ± 0.31	0.341	0.110

## Data Availability

The data presented in this study are available on request from the corresponding author. The data are not publicly available due to ethical restrictions.

## Abbreviations

The following abbreviations are used in this manuscript:

APB	Abductor Pollicis Brevis
ASA	Advanced Source Analysis
CED	Cambridge Electronic Design
CEM	Cervical Extensor Muscles
DCML	Dorsal Column Medial Lemniscus
DCN	Deep-Cerebellar Nuclei
EEG	Electroencephalography
EHI	Edinburgh Handedness Inventory
FMTT	Force-Matching Tracking Task
fMRI	Functional Magnetic Resonance Imaging
GABA	Gamma-Aminobutyric Acid
IFCN	International Federation of Clinical Neurophysiology
MTT	Motor Tracing Task
NMV	Neck Muscle Vibration
NV	No Vibration
PET	Positron Emission Tomography
SCM	Sternocleidomastoid
SCNP	Subclinical Neck Pain
SEPs	Somatosensory Evoked Potentials
SMI	Sensorimotor Integration
S1	Primary Somatosensory Cortex
TMS	Transcranial Magnetic Stimulation
UFT	Upper-Fibre Traps
V	Vibration
VPL	Ventro-Posterior Thalamus
